# Role of Citicoline in an *in vitro* AMD model

**DOI:** 10.18632/aging.103164

**Published:** 2020-05-29

**Authors:** Sonali Nashine, M. Cristina Kenney

**Affiliations:** 1 Department of Ophthalmology, Gavin Herbert Eye Institute, University of California Irvine, Irvine, CA 92697, USA; 2 Department of Pathology and Laboratory Medicine, University of California Irvine, Irvine, CA 92697, USA

**Keywords:** Citicoline, age-related macular degeneration (AMD), neuroprotection, RPE, mitochondria

## Abstract

Citicoline is the exogenous form of the nootropic, Cytidine 5'-diphosphate-choline that exerts its neuroprotective effects in the brain as well as in the eye. The current study characterized the cytoprotective effects of purified Citicoline in transmitochondrial AMD (Age-related Macular Degeneration) RPE cybrid cells which carry diseased mitochondria from clinically characterized AMD patients. The effects of Citicoline were examined via flow cytometry analysis of AnnexinV/ PI-stained cells, IncuCyte live-cell imaging analysis to quantify cells undergoing caspase-3/7-mediated apoptosis, analyses of gene expression profiles of apoptosis, hypoxia, and angiogenesis markers, and measurement of ROS levels and cell viability. Our results demonstrated that Citicoline when added exogenously alleviates apoptotic effects as evidenced by diminished AnnexinV/PI and Caspase-3/7 staining, downregulation of apoptosis genes, enhanced cell viability, and reduced oxidative stress in AMD RPE cybrid cells. In conclusion, our study identified Citicoline as a protector in AMD RPE cybrid cells *in vitro*. However, further studies are required to establish the merit of Citicoline as a cytoprotective molecule in AMD and to decipher the molecular underpinnings of its mechanism of action in AMD.

## INTRODUCTION

Citicoline is the international nonproprietary name given to the exogenous pharmacological form of Cytidine 5'-diphosphate-choline (CDP-Choline, CDPCho), a naturally occurring endogenous nucleotide compound that is water-soluble and has a molecular weight of 488.32 g/mol [1, 2]. CDP-Choline is comprised of cytosine base, ribose, pyrophosphate, and choline. The endogenous production of CDP-Choline from choline is an intermediate step in the *de novo* synthesis of phosphatidylcholine which is one of the most abundant cell membrane lipids in human and animal tissues [3]. By activating the biosynthesis of structural phospholipids, Citicoline maintains neuronal membrane integrity, influences neurotransmitter levels, increases norepinephrine and dopamine levels in the central nervous system, restores the activity of membrane sodium/potassium ATPase and mitochondrial ATPase, and enhances brain function [1]. Owing to these mechanisms, Citicoline has been successfully used as a neuroprotective agent to prevent neuronal aging and improve memory and learning *in vivo* [4]. Furthermore, it has been extensively used in preclinical studies and clinical trials for neurodegenerative diseases including Parkinson’s disease and glaucoma. Citicoline administration improves motor responses in Parkinson’s disease via stimulation of dopaminergic system [5]. Furthermore, Citicoline preserves the function of the retina and the visual cortex in glaucoma patients, and delays the progression of glaucoma disease [6–8]. Parisi et al. demonstrated that Citicoline injected intramuscularly improves retinal and visual function in glaucoma patients [9].

The primary advantages of Citicoline as a neuroprotective compound are: a) negligible toxicity in humans and animals, b) >90 % bioavailability, c) administration feasible via intravenous, intramuscular, or oral routes, and d) following oral ingestion, Citicoline is metabolized to cytidine and choline which enter the systemic circulation where cytidine is converted to uridine; both choline and uridine cross the blood-brain barrier [10–12]. Although the use of Citicoline in the rescue of neuronal cells and attenuation of retinal neurodegeneration is well-established, its potential role in preventing apoptotic cell death in retinal pigment epithelium (RPE) cells and in Age-related Macular Degeneration (AMD) pathology remains uncharacterized and awaits detailed investigation.

In quest of identifying novel therapeutic candidates for AMD, the goal of this study was to test the hypothesis that Citicoline, a naturally occurring nootropic, will protect against apoptotic cell death in an *in vitro* AMD model i.e., transmitochondrial AMD RPE cybrid cells which are created by fusing mitochondrial DNA-deficient APRE-19 (*Rho0*) cells with platelets isolated from AMD patients. Since nuclear content is the same and the cells differ only in mitochondrial DNA (mtDNA) content, the differences in biochemical or molecular profiles in AMD RPE cybrid cell lines can be attributed to variations in mitochondrial DNA of AMD patients. Our previous studies have shown that the AMD RPE cybrid cells carry mtDNA damage from the AMD patients. Extensive characterization studies using various endpoints that measure cellular and mitochondrial health have demonstrated dysfunctional AMD mitochondria, significantly higher mitochondrial superoxide generation, increased oxidative stress and apoptosis, and reduced mtGFP (Green Fluorescent Protein) staining in AMD RPE cybrids compared to normal RPE cybrids. Therefore, our previous findings have established substantive cellular damage due to increased oxidative stress and apoptotic cell death in AMD RPE cybrid cell lines compared to the normal RPE cybrid cell lines [13–15].

This *in vitro* study supports our hypothesis as Citicoline conferred significant protection against apoptotic cell death that was in-part mediated by damaged mtDNA from AMD patients in transmitochondrial AMD RPE cybrid cells.

## RESULTS

### Citicoline reduces apoptotic cells as shown by diminished Annexin V fluorescence intensity

**Figure 1:**
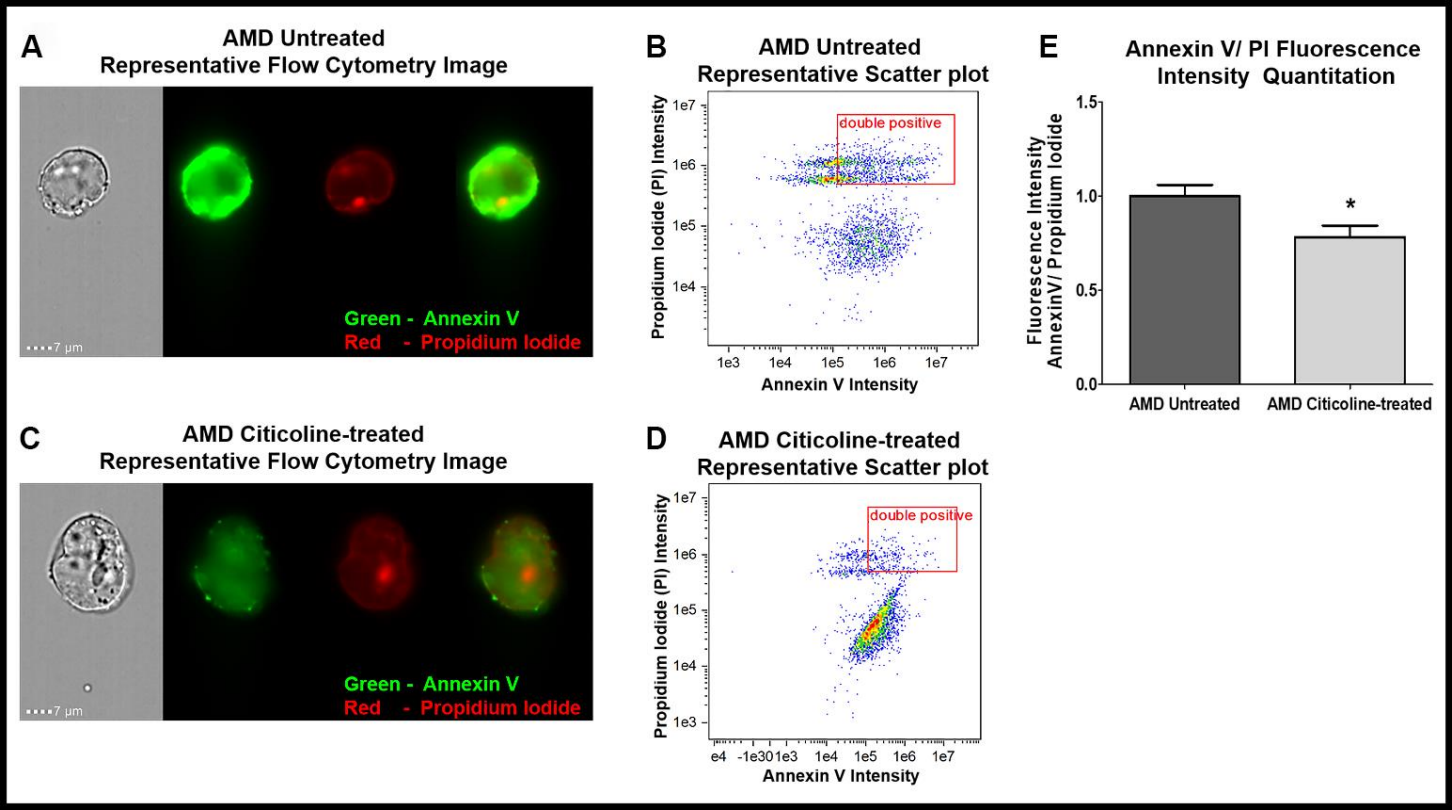
(**A**) AMD Untreated cells’ Representative Annexin V/ PI staining flow cytometry image; (**B**) AMD Untreated cells’ Representative Annexin V/ PI fluorescence intensity scatter plot; (**C**) AMD Citicoline-treated cells’ Representative Annexin V/ PI staining flow cytometry image; (**D**) AMD Citicoline-treated cells’ Representative Annexin V/ PI fluorescence intensity scatter plot; (**E**) AMD Untreated vs. AMD Citicoline-treated Annexin V/ PI fluorescence intensity quantitation.

The ability of Citicoline to attenuate apoptosis was examined via Flow Cytometry analysis of untreated and Citicoline-treated AMD RPE cybrid cells stained with apoptotic and dead cell markers, namely Annexin V and Propidium Iodide (PI), respectively (Figure 1A–1E). Figure 1A and 1C show representative Flow cytometry images and Figure 1B and 1D show representative scatter plots of untreated and Citicoline-treated AMD RPE cybrid cells stained with Annexin V/ PI.

Figure 1E quantifies the Annexin V/ PI fluorescence intensity in AMD RPE cybrid cells and demonstrates that Citicoline caused significant reduction in apoptotic cells. Flow cytometry analysis revealed a 21.67 % decrease in Annexin V/ PI double positives’ fluorescence intensity in Citicoline-treated AMD RPE cybrid cells (0.783 ± 0.06 a.u.) compared to their untreated counterparts (1 ± 0.059 a.u.) (p=0.04, n=6).

### Citicoline downregulates apoptosis-associated genes

**Figure 2:**
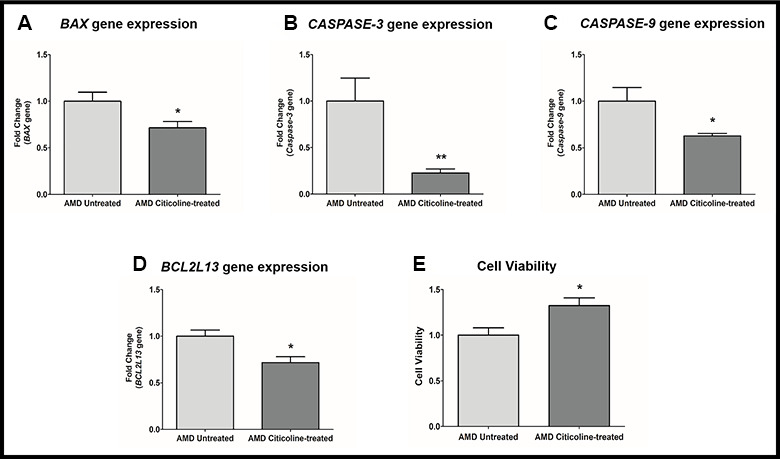
(**A**) *BAX* gene expression in AMD Untreated and AMD Citicoline-treated cells. (**B**) Caspase-3 gene expression in AMD Untreated and AMD Citicoline-treated cells. (**C**) Caspase-9 gene expression in AMD Untreated and AMD Citicoline-treated cells. (**D**) BCL2L13 gene expression in AMD Untreated and AMD Citicoline-treated cells. (**E**) Cell viability MTT assay.

Apoptosis is regulated by multiple genes that act at various levels of the apoptotic cell death pathway. Exogenous addition of Citicoline downregulated the pro-apoptotic genes significantly (Figure 2A–2D). Compared to their untreated counterparts, Citicoline-treated AMD RPE cybrid cells showed decreased gene expression of: *BAX* gene by 28.6 % (AMD Untreated: 1 ± 0.096, AMD Citicoline-treated: 0.714 ± 0.068; p=0.03, n=8) (Figure 2A), *Caspase-3* gene by 77.2 % (AMD Untreated: 1 ± 0.248, AMD Citicoline-treated: 0.228 ± 0.043; p=0.0079, n=5) (Figure 2B), *Caspase-9* gene by 37.2 % (AMD Untreated: 1 ± 0.147, AMD Citicoline-treated: 0.628 ± 0.028; p=0.03, n=5) (Figure 2C), and *BCL2L13* gene by 28.4 % (AMD Untreated: 1 ± 0.065, AMD Citicoline-treated: 0.716 ± 0.064; p=0.010, n=8) (Figure 2D). Furthermore, Citicoline treatment led to a 32.4 % increase in cell viability (AMD Untreated: 1 ± 0.081, AMD Citicoline-treated: 1.324 ± 0.084; p=0.015, n=6) (Figure 2E).

### Citicoline reduces Caspase-3/7-mediated apoptosis

**Figure 3:**
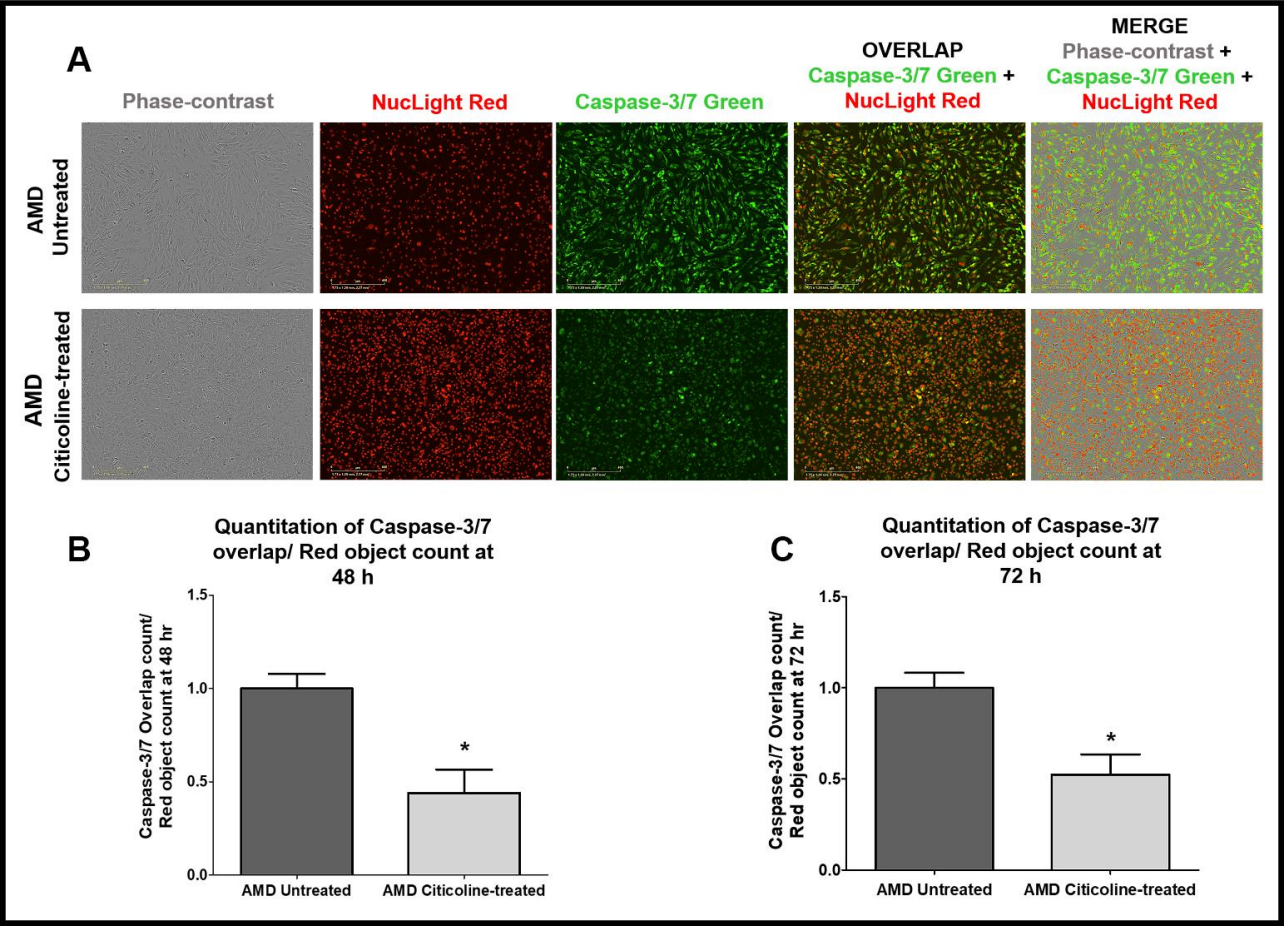
(**A**) Upper and lower panels show Representative Incucyte live-cell images of untreated and Citicoline-treated AMD cells ,respectively, in phase-contrast (first column), stained with NucLight Red (second column), stained with Caspase-3/7 Green (third column), overlap i.e., Caspase-3/7 + NucLight (fourth column), and Merge i.e., Phase-contrast + Caspase-3/7 + NucLight (fifth column). Scale bar = 400 μM.(**B**) Quantitation of Caspase-3/7 overlap/ Red object count at the 48 h time point. (**C**) Quantitation of Caspase-3/7 overlap/ Red object count at the 72 h time point.

To examine and compare Caspase-3/7-mediated apoptosis between untreated and Citicoline-treated AMD RPE cybrid cells, we performed IncuCyte® Live-Cell Imaging Analysis using Caspase- 3/7 Green and NucLight Red dyes (Figure 3A-3C). Figure 3A shows representative IncuCyte live-cell images. The upper panel represents untreated AMD group and the lower panel represents the Citicoline-treated AMD group. Addition of Citicoline led to a 55.99 % decrease in Overlap object count (i.e., Caspase-3/7 Green+NucLight Red staining)/ NucLight Red object count in AMD RPE cybrid cells: Untreated - 1 ± 0.078 a.u. and Citicoline-treated - 0.440 ± 0.125 a.u. (p=0.03, n=4) at 48 h (Figure 3B). At 72 h, a 47.54 % drop in Overlap object count was observed in Citicoline-treated AMD RPE cybrid cells (0.52 ± 0.11 a.u.) compared to their untreated counterparts (1 ± 0.082 a.u.) (p=0.03, n=4) (Figure 3C). Therefore, Citicoline prevents Caspase-3/7-mediated apoptosis in AMD RPE cybrid cells.

### Citicoline reduces oxidative stress

**Figure 4:**
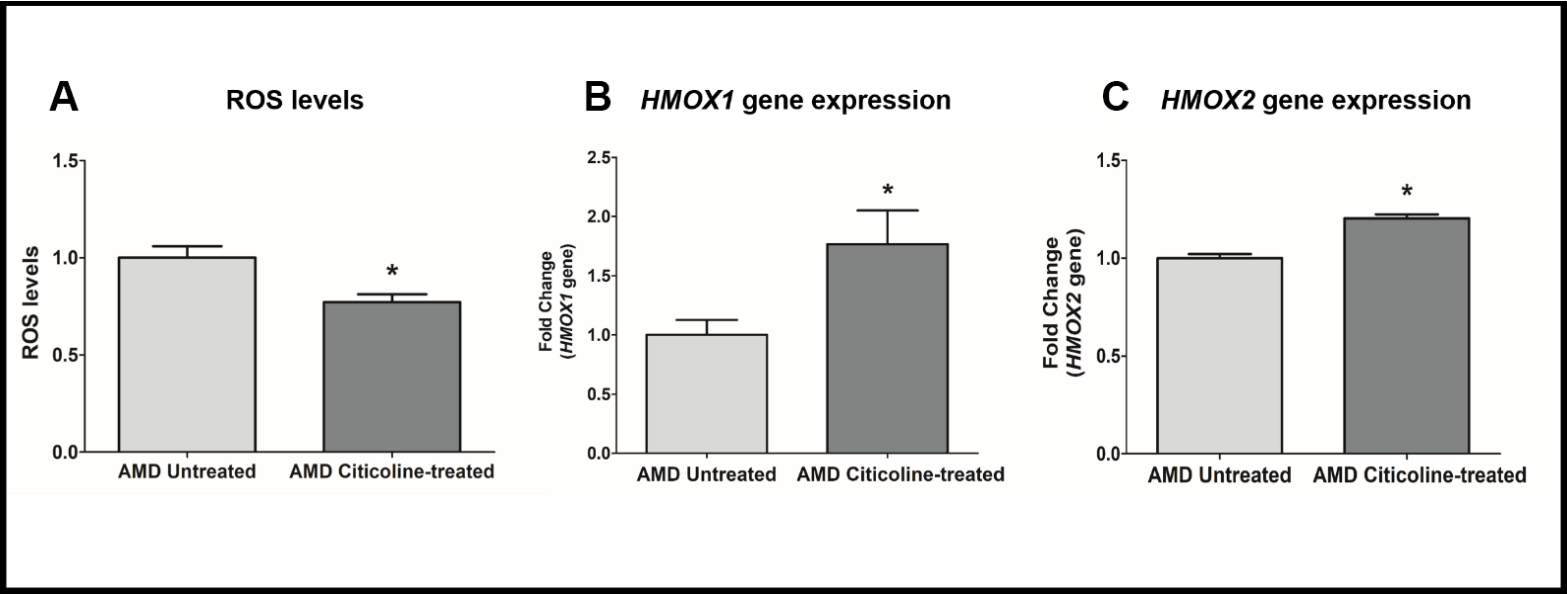
(**A**) ROS levels in AMD Untreated and AMD Citicoline-treated cells, (**B**) HMOX1 gene expression levels in AMD Untreated and AMD Citicoline-treated cells, and (**C**) HMOX2 gene expression levels in AMD Untreated and AMD Citicoline-treated cells.

To measure reactive oxygen species levels, we performed ROS assay using H2DCFDA reagent. Compared to their untreated counterparts, Citicoline-treated AMD RPE cybrid cells showed decreased ROS levels by 22.8 % (AMD Untreated: 1 ± 0.059, AMD Citicoline-treated: 0.772 ± 0.040; p=0.013, n=5) (Figure 4A). Compared to their untreated counterparts, Citicoline-treated AMD RPE cells showed increased gene expression of: *HMOX1* gene by 76.6 % (AMD Untreated: 1 ± 0.1267, AMD Citicoline-treated: 1.766 ± 0.28; p= 0.0379, n=8) (Figure 4B) and *HMOX2* gene by 20.4 % (AMD Untreated: 1 ± 0.0214, AMD Citicoline-treated: 1.204 ± 0.020; p=0.0286, n=4) (Figure 4C).

### Citicoline downregulates *HIF-1α* and *VEGF* genes

**Figure 5:**
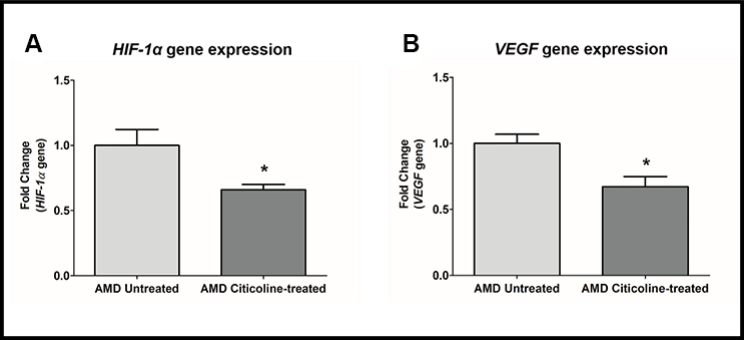
(**A**) *HIF-1α* gene expression in AMD Untreated and AMD Citicoline-treated cells. (**B**) *VEGF* gene expression in AMD Untreated and AMD Citicoline-treated cells.

*HIF1α* (Hypoxia-inducible factor 1-alpha), a transcription factor, is a master regulator of cellular response to hypoxic stress. *HIF-1α* activation leads to up-regulation of *VEGF*, which in turn plays a key role in angiogenesis in choroidal neovascularization in AMD. Compared to their untreated counterparts, Citicoline-treated AMD RPE cybrid cells showed decreased gene expression of: *HIF-1a* gene by 34 % (AMD Untreated: 1 ± 0.123, AMD Citicoline-treated: 0.66 ± 0.041; p=0.01, n=7) (Figure 5A) and *VEGF* gene by 32.8 % (AMD Untreated: 1 ± 0.069, AMD Citicoline-treated: 0.672 ± 0.077; p=0.015, n=6) (Figure 5B).

## DISCUSSION

Our current study identified the cytoprotective potential of exogenously added purified Citicoline in transmitochondrial AMD RPE cybrid cells *in vitro*. Using a combination of apoptotic assays, we found that Citicoline mitigates apoptotic cell death as evidenced by diminished Annexin V/ PI positive cell population, reduced Caspase-3/7-mediated apoptosis in live cells, downregulation of apoptotic genes, and enhanced cell viability in Citicoline-treated transmitochondrial AMD RPE cybrid cells. Additionally, treatment with Citicoline led to a significant reduction in reactive oxygen species and upregulation of *HMOX1* and *HMOX2* genes, thereby suppressing oxidative stress and supporting cell survival. Furthermore, significantly decreased expression of *HIF-1α* (hypoxia marker) and *VEGF* (angiogenesis marker) genes, post-Citicoline treatment, may in part have contributed to the cytoprotective action of Citicoline in AMD RPE cybrid cells. To our knowledge, this is the first report to identify the anti-apoptotic potential of Citicoline in an *in vitro* transmitochondrial AMD RPE cybrid cell model.

Apoptosis is characterized by specific morphological and biochemical changes in the cell, which can be detected via varied techniques. Annexin V is a eukaryotic cellular protein commonly used as a probe to detect apoptotic cells due to its ability to bind phosphatidylserine i.e., a cell membrane phospholipid that faces the cytoplasmic surface in healthy cells but is translocated to the extracellular side in apoptotic cells. Phosphatidylserine(s) exposure on the outer leaflet of the plasma membrane signals macrophages and marks the apoptotic cells for phagocytosis [16]. In this study, we used a recombinant Annexin V conjugated to the Alexa Fluor® 488 fluorophore to create a photostable conjugate with maximum sensitivity. Along with Annexin V, we used the red-fluorescent propidium iodide (PI) nucleic acid binding dye which is impermeant to live cells and apoptotic cells, but stains dead cells with red fluorescence. Flow cytometry analyses enabled us to distinguish viable cells from apoptotic cells and necrotic cells. In this study, Citicoline treatment led to diminished Annexin V/ PI fluorescence intensity, indicating the ability of Citicoline to lower apoptotic cell death in transmitochondrial AMD cells. This is consistent with a previous study in which the apoptosis inhibitory action of Citicoline was demonstrated using Annexin V/ FITC Flow cytometry analysis in a mouse model of cerebral malaria (CM); administration of Citicoline rescued cells in an experimental model of CM *in vitro* as well conferred partial protection against cell death and neurological syndrome in murine CM [17].

In the current study, Citicoline treatment in AMD RPE cybrid cells caused downregulation of *BAX*, *Caspase-3, Caspase-9*, and *BCL2L13* genes indicating that Citicoline mediates its cytoprotective effects by influencing both the intrinsic and extrinsic pathways of apoptosis. Our previous studies have demonstrated that dysfunctional AMD mitochondria in the AMD RPE cybrid cells contribute to the activation of apoptosis and enhanced expression of apoptotic markers such as *BAX* and *Caspase-3* [14]. BAX (Bcl-2-Associated X protein) is a member of the Bcl-2 family and a key regulator of the intrinsic apoptotic pathway. Apoptotic stimuli activate BAX and BAK (Bcl-2 homologous Antagonist/Killer) which oligomerize and initiate permeabilization of the mitochondrial outer membrane, which is considered a critical step in apoptosis [18]. Caspase-3 is an effector caspase that via its protease activity initiates and coordinates crucial apoptotic events such as the exposure of Phosphatidylserine to the extracellular side of the plasma membrane and cellular degradation processes including DNA fragmentation and cytoskeletal disruption. Caspase-3 is the point of convergence for the extrinsic and intrinsic apoptotic pathways [19]. On receiving apoptotic stimuli, the mitochondria release cytochrome c which binds to Apaf-1 and recruits Caspase-9 thereby activating the latter. Caspase-9 is a part of the apoptosome and initiates the activation of downstream effector caspases [20]. BCL2L13/Bcl-rambo is a member of the Bcl-2 family of proteins that regulate apoptosis. In cells, Bcl-rambo is localized to the mitochondria, and its overexpression induces apoptosis. Bcl-rambo mediates apoptosis by associating with adenine nucleotide translocator (ANT), a component of the mitochondrial permeability transition pore, to induce its opening [21]. Previous studies have attributed the Citicoline-mediated suppression of apoptosis to its ability to upregulate the Sirtuin1 (SIRT1) protein, downregulate procaspase and caspase expression, and neutralization of BAX family proteins thereby preventing cleavage of PARP and subsequent DNA damage [22–24].

Next, we compared Caspase-3/7-mediated apoptosis between untreated and Citicoline-treated AMD RPE cybrid cells using IncuCyte® Live-Cell Imaging Analysis system and Caspase- 3/7 Green and NucLight Red reagents. The IncuCyte Caspase-3/7 Green Apoptosis Reagent couples the activated Caspase-3/7 recognition motif (DEVD) to a DNA intercalating dye and enables real-time quantification of cells undergoing caspase-3/7 mediated apoptosis. This reagent is an inert, non-fluorescent substrate which when added to culture medium, crosses the cell membrane where it is cleaved by activated caspase-3/7 resulting in the release of the DNA dye and fluorescent staining of the nuclear DNA. The IncuCyte NucLight Rapid Red Reagent is a cell permeable DNA stain that specifically stains nuclei in live cells and enables real-time quantification of cell proliferation. Addition of this reagent to normal healthy cells does not interfere with cell growth and morphology and provides homogenous staining of nuclei. In the culture medium, this inert stain crosses the cell membrane and has excellent specificity for DNA without the need for a wash step. In the current study, Citicoline-treated AMD cells showed significantly lower Overlap object count (i.e., (Caspase-3/7 Green + NucLight Red staining)/ Red object count) at 48 h and 72 h compared to their untreated counterparts. To our knowledge, this is the first study to demonstrate the role of Citicoline in reducing Caspase-3/7-mediated apoptosis in live cell imaging systems.

Our current results are consistent with previous studies which have demonstrated the apoptosis inhibitory effect of Citicoline in various *in vitro* and *in vivo* models of neurodegenerative conditions. For instance, Alvarez et al. showed Citicoline-mediated protection of hippocampal neurons against apoptosis induced by brain beta-amyloid deposits plus cerebral hypoperfusion in rats [25]. Moreover, Citicoline protects against high-glucose-induced neurotoxicity and against excitotoxic cell damage in retina [26]. As demonstrated in recent studies, one mechanism by which Citicoline mediates its cytoprotective action could be via suppression of ERK1/2 signaling which is known to induce apoptosis in the inner and outer retina [27]. Additionally, Citicoline is known to exert it pro-survival action in diabetic retina by preventing glial activation and suppressing the expression of NF-κB and TNF-α [28].

The current study also revealed that Citicoline alleviates ROS production and downregulates *HIF-1α* and *VEGF* genes in AMD RPE cybrid cells. These results are corroborated by previous findings that demonstrate that Citicoline reduces ROS species, stabilizes cell membranes, reduces the volume of ischemic lesions, and provides neuroprotection in ischemic and hypoxic conditions via: a) attenuating the accumulation of free fatty acids especially arachidonic acid, b) preventing the activation of phospholipase A2 in both membrane and mitochondrial fractions, and c) stimulating the synthesis of glutathione [29, 30].

In summary, although further studies with Citicoline/ AMD RPE cybrid cells are underway, these results present novel findings that identify Citicoline to be a potential protector that attenuates apoptotic cell death in AMD. Citicoline is available as an over-the-counter dietary supplement in the U.S. and offers the advantage of easy access that shortens considerably the transition from lab bench to clinic.

## MATERIALS AND METHODS

### Human subjects

The University of California Irvine’s IRB (Institutional Review Board) approved research with human subjects (Approval #2003–3131). All participants provided informed consent and clinical investigations were performed according to the tenets of Declaration of Helsinki.

### Cell culture

Passage 5 AMD ARPE-19 transmitochondrial cybrid cell lines were created as described previously [14]. Briefly, these cybrid cell lines were prepared by polyethylene glycol fusion of mitochondria DNA-deficient ARPE-19 (*Rho^0^*) cell line with platelets isolated from AMD patients. Cybrid status and that the cybrids have acquired their mtDNAs from the donor individuals was confirmed using allelic discrimination, Sanger sequencing, and Next-Generation Sequencing.

### Culture conditions

The base medium for this cybrid cell line is DMEM-F12 Medium (Cat. # 10-092CM, Fisher Scientific, Pittsburgh, PA). DMEM-F12 Medium contains 3.15 g/L D-glucose, 2.5 mM L-glutamine, 15 mM HEPES, 0.5 mM sodium pyruvate, and 1200 mg/L sodium bicarbonate. To make the complete growth medium, fetal bovine serum was added to the base medium to a final concentration of 10 %.

### Treatment with Citicoline

Purified Citicoline was obtained from Sigma-Aldrich (St. Louis, MO) and used at a concentration of 1mM for all experiments. Water was used as an initial solvent. Citicoline was subsequently dissolved in culture media for treatment of cells.

### Flow cytometry

Cell were stained with recombinant Annexin V conjugated to fluorescein (FITC annexin V), as well as red-fluorescent propidium iodide (PI) nucleic acid binding dye (Life Technologies). The stained cells were analyzed by flow cytometry, measuring the fluorescence emission at 530 nm and >575 nm. Live cells show only a low level of fluorescence, apoptotic cells show green fluorescence and dead cells show both red and green fluorescence.

### Quantitative Real-Time PCR (qRT-PCR)

RNA extraction, cDNA synthesis, and qRT-PCR analysis were performed as described previously [14]. QuantiTect Primer Assays were used to study the expression of *Caspase-3* gene (Cat. # QT00023947, Qiagen, Germantown, MD), *BAX* gene (Cat. # QT00031192, Qiagen), *HIF-1α* gene (Cat. # QT00083664, Qiagen), *HMOX1* gene (Cat. # QT00092645, Qiagen), and *HMOX2* gene (Cat. # QT00039942, Qiagen). KiCqStart® SYBR® green primers were used to examine the expression of *VEGF* gene (Cat. # kspq12012, Sigma). Specific housekeeper gene used was *HPRT1* (Cat. # QT00059066, Qiagen). Data analysis was performed using ΔΔCt method which was calculated by subtracting ΔCt of the AMD group from ΔCt of the normal group. ΔCt was the difference between the Cts (threshold cycles) of the target gene and Cts of the housekeeper gene (reference gene). Fold change was calculated using the following formula: Fold change = 2^ΔΔCt^.

### Cell viability assay

The numbers of viable cells were measured using the MTT (3-(4,5-dimethylthiazol-2-yl)-2,5-diphenylte-trazolium bromide) assay. Cells were plated in 96-well tissue culture plates, treated with 1 mM Citicoline followed by addition of MTT. Cells were incubated at 37 °C for 1 h, followed by addition of DMSO (DiMethyl SulfOxide). Signal absorbance was measured at 570 nm and background absorbance measured at 630 nm. Normalized absorbance values were obtained by subtracting background absorbance from signal absorbance. The colorimetric signal obtained was proportional to the cell number.

### IncuCyte live-cell imaging

IncuCyte live-cell imaging was performed as described previously [31, 32]. Cells were seeded in 96-well plates at a density of 5,000 – 10,000 cells/well followed by staining with IncuCyte® NucLight Rapid Red (1:500) and Caspase-3/7 Green (1:1000) labeling reagents. Stained cell plates were placed into the IncuCyte® live-cell analysis system and allowed to warm to 37 °C for 30 min prior to scanning. Phase Contrast, Green, and Red channels were selected, 5 images were taken per well with an average scan interval of 2 h until the experiment was complete. Fluorescent objects were quantified using the IncuCyte® integrated analysis software that minimizes background fluorescence.

### Reactive oxygen species (ROS) assay

To quantitate ROS levels, the cell-permeant H2DCFDA (2', 7’-dichlorodihydrofluorescein diacetate) was used as an indicator for ROS in cells. Stock solution of 5mM H2DCFDA was prepared in DMSO. Stock solution was then diluted in DPBS (Dulbecco's Phosphate-Buffered Saline) to obtain a working concentration of 10 μM. Cells were plated in 96-well tissue culture plates followed by treatment with 1mM Citicoline. 10 μM H2DCFDA solution was added to cells and incubated for 30 min at 37 °C. H2DCFDA was then replaced with DPBS. Fluorescence which was measured at excitation 492 nm and emission 520 nm was proportional to ROS levels in cells.

### Statistical analysis

Non-parametric Mann-Whitney test (GraphPad Prism 5.0; GraphPad Software, CA, USA) was used to analyze data between groups and to determine significance; p ≤ 0.05 was statistically significant. ‘n’ represents the number of biological replicates i.e., the number of individual AMD cybrid cell lines used in the experiment.
